# Sensor-Based Safety Performance Assessment of Individual Construction Workers

**DOI:** 10.3390/s18113897

**Published:** 2018-11-12

**Authors:** JeeWoong Park, Yong K. Cho, Ali Khodabandelu

**Affiliations:** 1 Department of Civil and Environmental Engineering and Construction, The University of Nevada, Las Vegas, NV 89154, USA; khodaban@unlv.nevada.edu; 2 School of Civil and Environmental Engineering, Georgia Institute of Technology, Atlanta, GA 30332, USA; yong.cho@ce.gatech.edu

**Keywords:** construction, safety, awareness, communication, sensing

## Abstract

Over the last decade, researchers have explored various technologies and methodologies to enhance worker safety at construction sites. The use of advanced sensing technologies mainly has focused on detecting and warning about safety issues by directly relying on the detection capabilities of these technologies. Until now, very little research has explored methods to quantitatively assess individual workers’ safety performance. For this, this study uses a tracking system to collect and use individuals’ location data in the proposed safety framework. A computational and analytical procedure/model was developed to quantify the safety performance of individual workers beyond detection and warning. The framework defines parameters for zone-based safety risks and establishes a zone-based safety risk model to quantify potential risks to workers. To demonstrate the model of safety analysis, the study conducted field tests at different construction sites, using various interaction scenarios. Probabilistic evaluation showed a slight underestimation and overestimation in certain cases; however, the model represented the overall safety performance of a subject quite well. Test results showed clear evidence of the model’s ability to capture safety conditions of workers in pre-identified hazard zones. The developed approach presents a way to provide visualized and quantified information as a form of safety index, which has not been available in the industry. In addition, such an automated method may present a suitable safety monitoring method that can eliminate human deployment that is expensive, error-prone, and time-consuming.

## 1. Introduction

Over the last decade, researchers have explored various technologies and methodologies to enhance the safety of workers at construction sites. Regardless of the methods used, a holistic approach to improving safety should be based on continuous monitoring of the construction site to detect potentially unsafe conditions/hazardous events. However, the complex environment of indoor construction sites and continuous changes in daily activities often lead to difficulty in conducting safety inspections by site managers [1,2,3,4]. In addition, their method for conducting these inspections relies on manual observation, which is inefficient, labor intensive, prone to error, inconsistent, and costly [5,6,7,8]. 

Insufficiently identified safety issues may result in potentially hazardous events that may escalate to injuries and fatal accidents. Even though the construction industry has adopted safety training and regulations to enhance worker safety, safety issues have continued to threaten workers’ health and lives, and have become a significant problem. Statistics from various organizations indicate that the accident rate of the construction industry ranks among the highest among private industries in the USA [9,10].

Researchers have explored using sensing technologies that can potentially benefit the construction industry in various aspects [8,11,12]. For example, real-time location systems (RTLSs) have been developed to monitor and collect real-time information from a site [13,14,15,16,17,18,19]. As of yet, however, little research has been done to explore the issue of individual workers’ safety by using RTLSs, and a holistic and integral approach has not been developed. To address this challenge, this paper introduces a zone-based safety risk model that quantifies the safety performance of individual workers based on a previously developed RTLS [20,21].

## 2. Background

Continuous monitoring of a construction site is crucial to provide workers with a work-friendly environment that presents minimal hazards to their health and safety. In an effort to enhance safety, the construction industry has adopted several methods, such as accident investigations, self-inspections, surveys, and job hazard analyses. However, these are passive methods of data collection because they require site observations or they are created after the undesired events already occurred; therefore, all incidents that have the potential to lead to accidents that may not have necessarily been captured.

When monitoring and identifying safety-related occurrences, the construction industry has relied heavily on manual efforts [22,23,24], such as data of past safety performance, which are recorded manually after the occurrence of an event. These recorded data provide value in understanding the issues and safety trends of the construction activities, but they require such steps as manual data collection, aggregation, and postanalysis. Although such a method produces a project/company level of safety information, which is still valuable, it is difficult to extract safety information for individual workers from such a complex process.

For certain tasks, Occupational Safety and Health Administration (OSHA) requires the designation of a competent person for safety purposes. This person should be able to identify existing and predictable hazards at the site and should have the authority to take actions to eliminate such hazards [25]. In recent years, monitoring of the safety conditions of workers has become more challenging with the increasing complexity of construction projects. Because of this trend in construction, safety managers are challenged with continuously monitoring and identifying incidents that may cause safety problems, and their ability to accomplish this task and to make proper and prompt decisions may be inadequate, in certain cases [26]. Furthermore, this limited capability of the safety managers may cause some difficulty with regard to the need for ubiquitous and continuous on-site monitoring for the precise identification of construction safety issues [27,28,29].

As a result, near-miss events—that is, incidents that could potentially escalate into an accident—are often ignored or neglected by associated personnel, and are not properly recorded [30,31]. Li et al. [32] pointed out that the number of near-miss incidents are considerably greater than the number of accidents that are actually recorded. Previously explored methods [12,33] can only quantify safety incident data in an on-and-off-based (or only alert based) metric, without having an ability to describe quantitatively dangerous incidents that actually do not result in an accident. Unfortunately, all near-miss events, which may escalate into accidents, could result in significant damage not only to the associated person but also to the associated contractors. One recent study [12] developed a tracking system to capture near-miss events, and Isaac and Edrei [33] advanced safety research forward by presenting a statistical model as well as providing more proactive alerts for increased risk exposures. These methods are advanced automated safety monitoring, and are effective in detecting safety violations in a discrete manner. Despite the advancement made by past research, worker safety performance is still difficult to understand in a simple, quantitative format. Therefore, for efforts in promoting a safe construction environment, construction safety practices/research are still inadequate, and the industry lacks an important measure for quantitative evaluation when handling safety incidents. In addition, measures used in practice tend to be subjective, resulting in a different conclusion each time, and from person to person. This study investigates a sensor-based safety monitoring approach in order to overcome this challenge by implementing a computational and analytical procedure/model. Through the developed system, the continuous monitoring and collection of a data stream from a construction site should be implemented so that detecting unsafe conditions or hazardous events is possible. The developed framework or analysis procedure should be available to process such data, using mathematical models to generate information that is quantitatively meaningful. As a byproduct of these models, an objective safety evaluation will follow.

## 3. Objective and Scope

Despite available sensing approaches and concerns on worker safety, a large gap among sensor data, modeling of safety issues, and individuals’ safety performance existed. This gap has not been properly investigated, and unfortunately, individuals’ safety performance remains poorly understood. Therefore, the objective of this research was to develop a sensor-based safety monitoring method. This involved defining and developing parameters for zone-based safety risks, and establishing a procedural model to quantify the zone-based safety risk (ZBSR) to individual workers. The ZBSR model uses a tracking system based on Bluetooth Low Energy (BLE) that was developed in previous studies [20,21]. The ZBSR model aims to mathematically process real-time location data to produce measures that could assist in the understanding of the behavior of workers, which are represented by safety performance indices that are computed based on locational information about identified hazards and their associated parameters, such as the hazard boundary (e.g., core and envelop zones) degree of exposure, frequency of exposure, and potential degree of injury. This procedure serves as an objective, quantitative method to evaluate safety performance determined by data collected onsite. To assess the module of the automated safety performance analysis, field experimentations were conducted in a controlled setting.

The scope of this research (i.e., zone-based safety risks) included spatiotemporal hazards predefined by zones and the workers’ interactions with these zones. According to the Health and Safety Executive Annual Statistics Report [34], over 20% of fatal accidents in the construction industry are associated with workers moving through zones at a construction site. Accidents also occur to workers while they are executing their tasks in a nonhazard zone. However, because the direct causes of such accidents vary, requiring a unique handling method for each cause, the scope of this study was limited to zone-related hazards that can create dangerous situations to workers. These zone-based hazards include, but are not limited to, hazards associated with the physical conditions of a construction site—for example, unprotected large openings—which accounts for 38% of the incident cases [35]. Such hazards are represented by their spatial and temporal relationship and the type of construction activities, if any are nearby. The scope did not include other hazardous events that could occur to workers while they are working at nonhazardous zones, such as cutting fingers, falling from a ladder, mistakes when operating equipment, electrocution by mistake, and others.

## 4. Method

The approach using ZBSR for individual workers was developed by

1. Establishing hazard models,
2. Identifying the exposure relationship between workers and associated hazards,
3. Formulating a quantitative relationship between the associated hazards and modeling parameters, and
4. Incorporating all of the parameters to compute an index that represents the safety performance of the worker.

The following subsections describes these processes.

### 4.1. System Design

**Figure 1:**
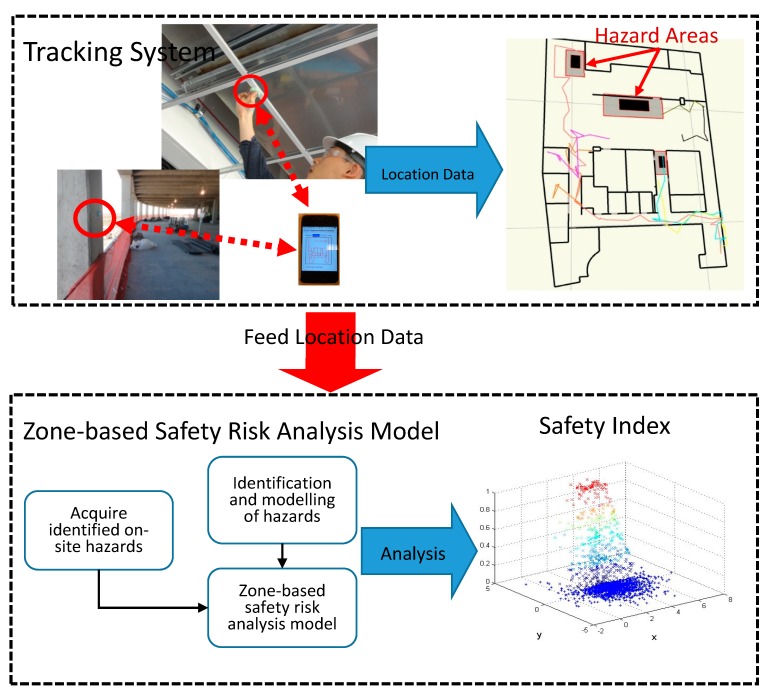
Flowchart of the automated safety monitoring system developed in this study.

To develop a comprehensive safety monitoring system, this paper first introduces the system design to establish the ZBSR model that uses real-time location data of workers onsite. Figure 1 displays a flowchart for the automated safety monitoring system, which integrates the tracking system with the ZBSR model. This integration allowed the evaluation of the safety performance of individual workers based on their location data collected by the tracking system. As discussed previously, only a little research has explored the use of RTLS in assessing the safety conditions of individual workers. Therefore, the remaining subsections in methodology focused on the development of a safety assessment approach that utilizes a real-time tracking system for quantifying the safety performance of individual workers with respect to various parameters.

### 4.2. Hazard Registration and Model

The safety performance of workers was assessed with respect to previously identified hazards (which can be available by pre-project planning, daily site inspection, and training). Hazard identification is typically carried out in two ways. By scrutinizing project information together with building information modeling (BIM) and work schedules under hazard detection rules, certain hazards could be identified automatically [7,36]. These types of hazards are often pre-identified hazards as they can be found by analyzing associated project information. Unlike these types of hazards, there also exist hazards that cannot be identified automatically. These types of hazards usually reflect specific project or site information that could change over time as the work progresses. Examples of these spatiotemporal hazards include poorly maintained areas, such as poor housekeeping areas, inappropriately piled stock areas, broken barricades, and scaffolds that violate safety rules.

Upon identification, hazards need to be modeled with certain parameters that quantitatively define the hazards. Such parameterized hazard models allow the evaluation of the safety conditions of workers with respect to the hazards. Each hazard varies with respect to type, size, and potential consequences; therefore, modeling of these hazards needs to account for these factors.

To describe the hazards in a unique manner, the ZBSR model used a safety envelope approach based on previous research [37,38,39]. Hazards that were defined using this approach provided information regarding the core hazards and the hazard envelope with respect to certain geometric information, such as radius, width, and length. The core hazard was represented by a zone that must not be breached, and the hazard envelope was represented by a zone that should be protected. The ZBSR model followed the same classification of hazards as found in a past paper [12]; any breach into the core hazard zone was considered as an imminent hazard, and any breach into the hazard envelope was considered as a caution event. All imminent hazards do not necessarily lead to an accident, but they should be noted since they indicate a clear violation. This parameter can be predefined based on hazard types and automatically parameterized in the system when the site manager has identified site hazards. For example, one of the leading causes of occupational injuries and fatalities are falls from portable ladders. As a means of protection, OSHA suggests erecting a barricade around the ladder being used in order to keep traffic away from the ladder [40]. Such a hazard was modeled by certain geometric shapes, such as a circle or an ellipse. Other types of hazards were modeled by a rectangular shape, for example, large penetrations (i.e., large holes), storage areas for hazardous material, restricted areas, and unsafe work zones.

**Figure 2:**
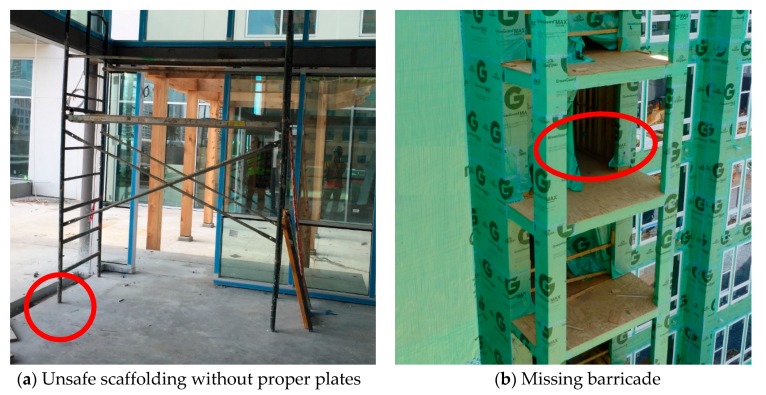
Hazards identified on a specific day for (**a**) a scaffolding hazard and (**b**) a ladder hazard.

**Figure 3:**
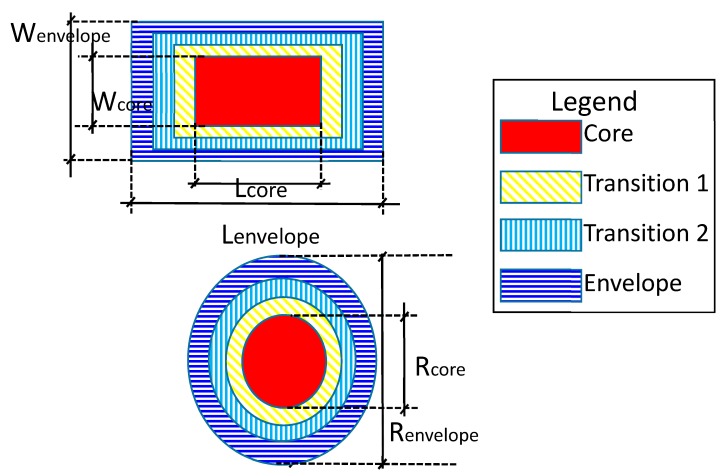
Parametric modeling of hazard severity.

Figure 2 shows examples of such hazards. The scaffolding hazard in Figure 2a is a type of hazard that can be identified through onsite inspection, and the ladder hazard in Figure 2b is a type of hazard that can be identified by project information analysis. The modeling of hazards to define geometric information would be up to the user’s discretion (e.g., the safety manager or engineer). Depending on the need for a detailed envelope zone, the user can set the geometric parameters of the envelope zone from 0 to a specific range, the case of 0 being a hazard that was detected by on-and-off violations. Figure 3 shows the parametric modeling of two types of hazards that have different geometric shapes that eventually were fed into the ZBSR analysis model for safety performance assessment.

### 4.3. Evaluation Metrics

Besides the parameters, which model existing hazards with respect to the discussed aspects, in order to assess individual safety performance based on their location data by using hazard models, the assessment also should account for dynamic location data in a solid relationship with the hazards. The solid relationship should offer a guide for quantitative assessment of the safety conditions of the worker by evaluating such parameters as the exposure level, exposure frequency, and the degree of potential damage or injury upon occurrence of an accident. The quantification of such parameters enables an objective assessment of the safety performance of workers in a systematic way.

The quantification of safety performance required concrete criteria and rules for assessing the safety conditions of workers, based on given information. The first metric was the degree of danger to a worker given the hazard models and the location data. It is evaluated based on the identified hazard zones and the location data of workers tracked by a tracking system. For example, if the worker was within the hazard envelope, it was considered a caution event, and the associated degree of danger was computed based on the equation developed in the following sections. To further detail out the quantification, a rule or a criterion using this geometric information of hazards was necessary in order to evaluate the degree of danger to a worker when the worker was exposed to any of the identified hazards. The rule needed to reflect the level of proximity in a relationship; this implies that the closer the worker was to the core hazard, the greater the chances that the worker would be involved in an accident.

**Figure 4:**
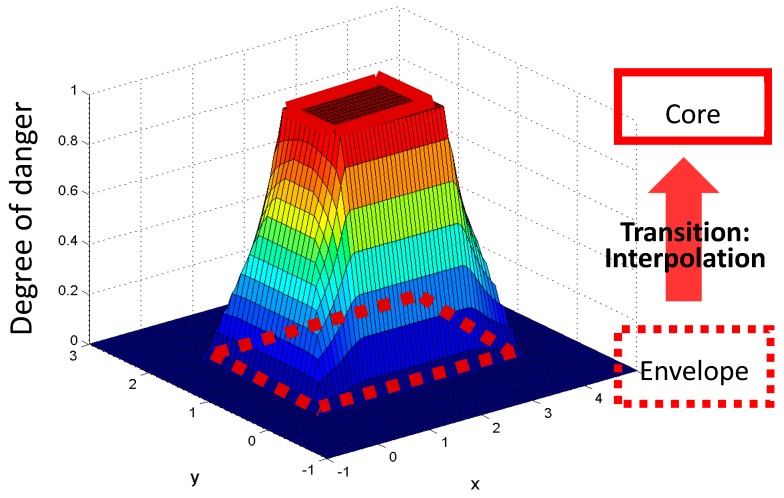
Linear modeling in detail of the degree of danger of a particular hazard.

Figure 4 displays a three-dimensional linear model for the degree of danger for a rectangular-shaped hazard. Although a linear model was used in this research, the type of model is adaptable in the proposed framework. Thus, depending on the safety manager’s discretion, or based on historical data, the type of model of the degree of danger could be adjusted. This model assumed that any breaches into the core zone was a critical event, whether it led to an accident or not; thus, all of such breaches were noted with a ‘high impact’ score of 1. However, if the worker was found to be outside of a hazard envelope, that situation was considered safe, and given a score of 0. The intermediate zone between the core and envelope zones was measured by linear interpolation. The same rules applied to other cases of hazard models. With this evaluation model, the uncertainty of the location data was assessed quantitatively even further during the analysis.

Each construction hazard presented various levels of danger; for example, ‘a-fall-to-a-lower-level’ accident likely involves more serious damage to the workers affected than does ‘a-trip-accident’. Despite this difference in potential damage, the method of introducing a safety envelope may not sufficiently cover the consequences caused by its potential damage. To take this into account, the ZBSR approach used a scaling factor to intensify the degree of danger. As this serves to estimate the potential consequence to workers associated with the hazard, historical data is a good resource to define the factor (e.g., if a-trip-accident is considered as a normal hazard having a scaling factor of 1, then a-fall-to-a-lower-level can be considered as a significantly dangerous hazard having a scaling factor of 2). The procedure generated an index that indicated the rate of the occurrence of an accident with respect to a specified time interval (e.g., per day). Because it is a representation of the degree of danger at a certain time interval, and because the safety monitoring system continuously collects and generates such data over a period, the system aggregated all of the data and produced a safety performance index in various forms that depended on the inspector’s needs. 

### 4.4. Zone-Based Safety Risk Analysis Based on RTLS Data

The ZBSR analysis occurred after the hazard modeling as well as the evaluation rules and criteria were completed. This included incorporation with a feed of real-time location data from on-site workers to complete the development of the quantitative procedure for the ZBSR analysis. In this approach, contextual data (e.g., worker information and location information), which was collected on-site, was translated into a quantitatively meaningful index that represented the safety condition of individual workers. The translation factored in an understanding of the workers’ safety-related behaviors and conditions. The associated parameters in the ZBSR analysis might not be quantified deterministically because of the uncertainties involved; for example, assumptions when modeling of parameters might lead to model uncertainties and quantified data might contain measurement uncertainties. To account for variability and uncertainties, the ZBSR model used a probabilistic approach to combine the parameters and input data in order to evaluate the safety performance of a worker.

Regarding a general overview of the equations associated to ZBSR, the quantification of safety performance involved the various parameters discussed, expressed as:

(1) $$spi_{i,j}=f\left(loc_{i},\;haz_{j},\;exp_{i},\;scale_{j},\;freq\right)\;\mathrm{for}\;a\;\mathrm{given}\;\mathrm{hazard}\;\mathrm{and}\;\mathrm{location},$$

where:

*spi_i_* = safety performance index for given location, *loc_i_* and the *j*th hazard
*loc* = location of position estimate
*haz_j_* = hazard models for the *j*th hazard
*exp_i_* = exposure level/degree of danger for given location, *loc_i_*
*scale_j_* = scale factor for the *j*th hazard
*freq* = frequency/exposure time

As the location estimation by the tracking system was not deterministic, the position estimation was evaluated probabilistically with regard to its accuracy, based on the standard deviation of the system as expressed as

(2) $$loc_{i}=f\left(x_{i},y_{i};\;x_{est},y_{est},\;std\right)$$

where,

*x_i_*, *y_i_* = actually possible positions
*x_est_*, *y_est_* = position estimation from the system
*std* = standard deviation of the position estimation

The ZBSR analysis used a normal distribution for generating candidate particles (*x_i_*, *y_i_*), given the location estimation (*x_est_*, *y_est_*) and the standard deviation.

Equation (1) was written for a given hazard, or the *j*th hazard. Two types of hazard models and their associated parameters that describe the hazards are expressed as

(3) $$haz_{j}=\{\begin{array}{c} f\left(length_{core},\;width_{core},\;length_{envelop},width_{envelop}\right)\\ f\left(radius_{core},\;radius_{envelop}\right) \end{array}\;\mathrm{for}\;j\mathrm{th}\;\mathrm{hazard}$$

For computing the exposure level, the necessary parameters are

(4) $$exp=\underset{all\;i}{\sum }exp_{i}=\underset{all\;i}{\sum }f\left(x_{i},y_{i},haz,\;scale\right)=f\left(prox1,\;prox2,\;scale\right)$$

where,

*prox*1 = the distance to the edge of the hazard core for a given hazard
*prox*2 = the distance to the edge of the hazard envelope for a given hazard

When computing the exposure level, the equation needs to be checked for all possible locations for a given location with uncertainty, all identified hazards located nearby, and continuous time. For simplicity, Equation (4) shows the computational formula for one hazard (i.e., no *j* term) and an instant time (i.e., no *k* term). ZBSR first found the distances from the worker’s claimed location (*x_i_*, *y_i_*) to the closest point of a hazard core (*prox*1) and that of a hazard envelope (*prox*2). It then used a linear interpolation to quantify the degree of danger, also known as the exposure level. 

Given a location datum point of a worker (i.e., the location estimation indicated by *x_est_* and *y_est_*) at a specific time interval, the ZBSR model checked all of the nearby hazards to comprehensively assess the safety performance. A general integral method that computes the safety performance index with a given location estimation and its uncertainty was used, and is expressed as

(5) $$y=\int \int \int y_{{\left(i,j\right)}_{k}}=\int \int \int f{\left(loc_{i},\;haz_{j},\;exp_{i,j},\;scale_{j},\;freq\right)}_{k},$$

where *y* is the safety performance index by ZBSR. Note that the frequency term has an additional term *k* to account for the evaluation in continuous time:

(6) $$y=\sum_{all\;k}\;y_{k}=\sum_{all\;i}\sum_{all\;j}\sum_{k}\;f{\left(loc_{i},\;haz_{j},exp_{i,j},\;scale_{j},\;freq\right)}_{k},$$

where *j* is the index for hazards and *k* is the index for time.

However, the integral in the safety performance equation is a continuous function over time, defined by the *k* term. Because of the complexity in solving this continuous integral with discrete data, Equation (5) instead was modified to a numerical summation so that the assessment was made in a discretized manner, as shown in Equation (6). In the discretized version of the assessment, index *j* covered the situation where the worker was involved with more than one hazard, and index *k* aggregated the safety performance evaluations that were continuously generated as the worker continued movements. The system yielded the corresponding safety evaluations.

## 5. Experiment and Result

The experimental test involved two sets of field experimentations to test the ZBSR models by quantifying the safety performance of a worker who was exposed often to hazardous areas. For safety reasons, this test was conducted in a controlled environment with trained subjects, and emulated certain safety incidents and violations that could control the safety conditions of the site. A controlled movement, which served as ground truth, provided a benchmark for comparison with the performance results acquired by the proposed approach. 

**Figure 5:**
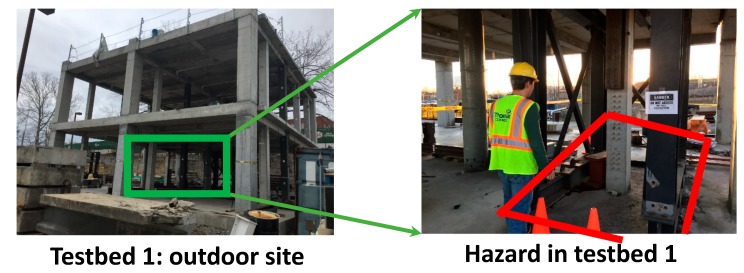

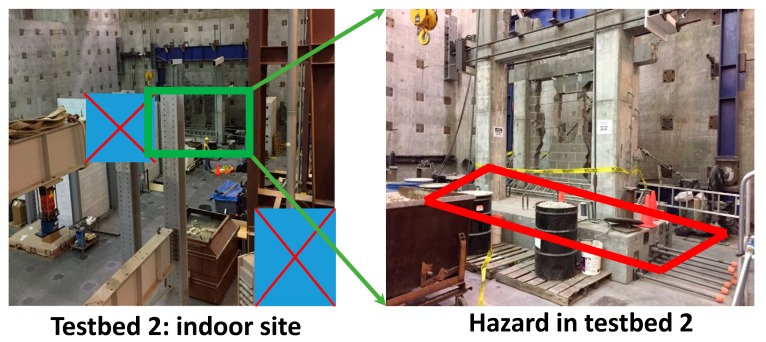
Two testbeds and their hazardous areas.

Figure 5 shows the two test beds and the associated hazard areas. This validation assumed a locational accuracy of approximately 1.5 m, which was concluded from the author’s previous studies with a BLE-based location tracking system [20,21]. As used in past work [12], BLE sensors were laid out over the site with an interval of 5 m. This system offered a sampling rate of 0.7 data per second. The tracking data were collected and analyzed with respect to the pre-identified hazards. This accuracy was used as the uncertainty input when processing the ZBSR model for quantifying the safety performance of a test subject. Detailed information associated with the tracking system can be found in the authors’ previous work [20,21]. The framework developed in this research used the accuracy of a tracking system as an input to the safety evaluation system, and should work for any tracking system in the same manner.

**Figure 6:**
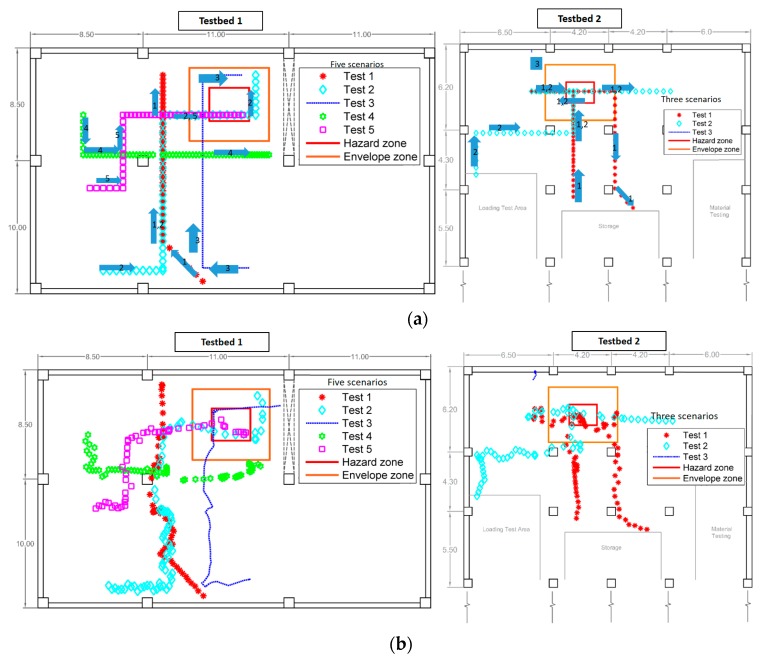
Tested scenarios in two test sites: (**a**) ground truth data and (**b**) tracking results.

To create various cases that represent a range of degrees of the level of proximity, exposure time, and exposure frequency, the study designed a multitude of scenarios for each of the two testbeds. Figure 6 shows the scenarios, which were designed such that the projected positions were located in various locations (core, transition, and envelope) within a hazard. The size of each imminent hazard zone was specified by the site manager, based on the space and conditions of the hazard. The size of the corresponding caution was chosen to be twice as large as that of the imminent hazard [12] that the safety manager considered reasonable. Based on these scenarios, the subject passed through a hazard zone and/or stayed in/out of a hazard zone. The tracking system collected the location information of the subject. Then, the ZBSR model was applied to interpret and analyze the data in order to assess the safety performance of the subject in the form of a safety index.

**Figure 7:**
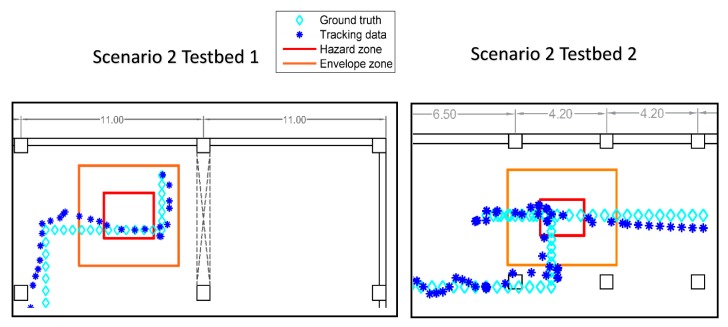
Zoomed-in view of tracking results and the corresponding ground truths of certain test cases.

Figure 7 shows two samples (i.e., Scenario 2 in Testbed 1 and Scenario 2 in Testbed 2) of tracking results and corresponding ground truths; the arrows indicate the direction of movement in the paths. Note that in actual application, the system does not know ground truth, but has to rely solely on the tracking data, which justifies the adoption of a probabilistic approach in our framework. Once the tracking data were collected, the system fed them into the ZBSR model for analysis. Because of the uncertainties as discussed previously, the safety performance was assessed probabilistically by applying Equations (1)–(6). ZBSR first received the streaming of the position estimation—that is, it takes each of the estimated points individually into the analysis—and associated the estimation with the hazard models registered in the system. After processing the position data by using Equations (1)–(6), the probabilistically assessed safety performance is generated.

**Figure 8:**
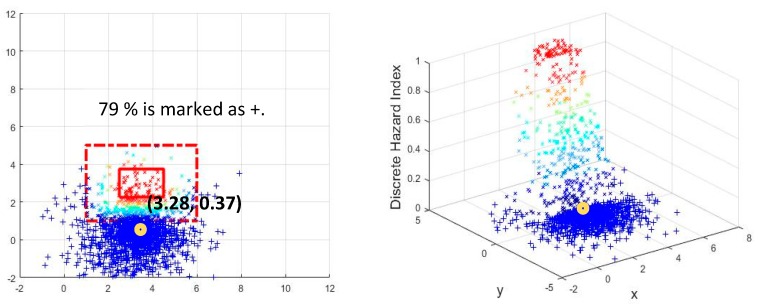

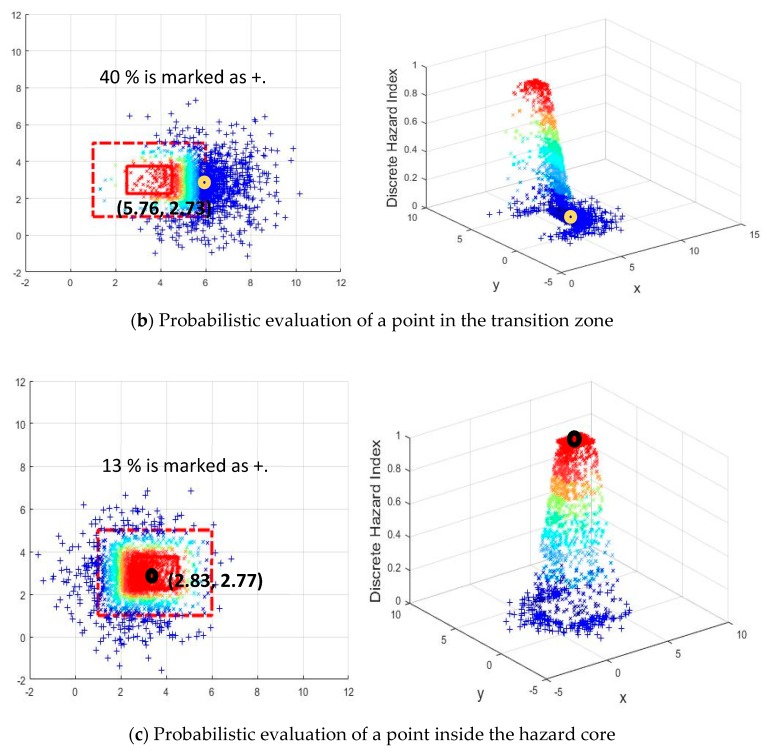
Evaluation of sample points from Scenario 2 of Testbed 2.

The authors selected three sample points for illustration purposes (see Figure 8), these three points were selected from Scenario 2 in Testbed 2 that best describe the situations. The first point, shown in Figure 8a, represents the case of safety assessment when the subject is near the hazard zone but does not invade the zone. The second point, shown in Figure 8b, represents the case of safety assessment when the subject is in the hazard core. The third point, shown in Figure 8c, represents the case of safety assessment when the subject is inside the hazard envelope but outside the hazard core. As observed, the number of points in each of the hazard zones seems reasonable because the decreasing number of + marked points from the case in Figure 8a–c properly reflects the increasing exposure level, and the numbers of + marked points are 79%, 40%, and 13%, respectively. The right-hand plots for each case show a 3D evaluation of the hazardous degree of each of the (*x*, *y*) points, based on the linear model. The plots yielded congruent results with previous observations. As the position estimation advanced towards the core of the hazard zone, the number of points on a high scale of increase.

**Figure 9:**
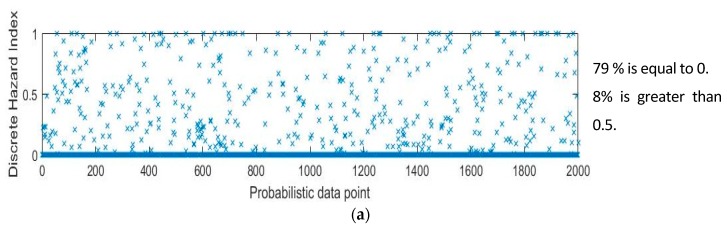

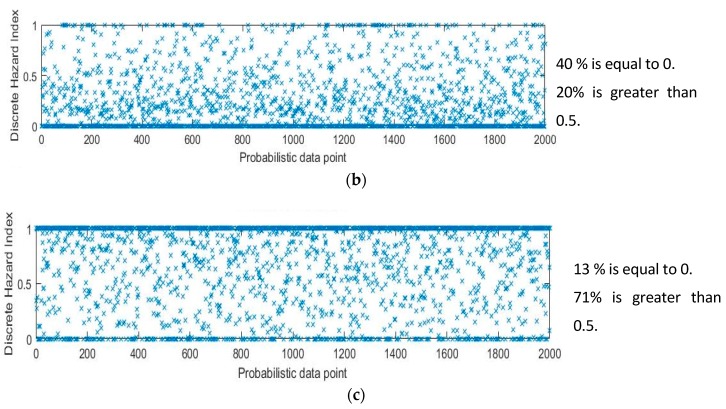
Detailed assessment of each of the evaluations. (**a**) 2D evaluation of a point (3.28, 0.37) outside the hazard zone (corresponding to Figure 8a); (**b**) 2D Evaluation of a point (5.76, 2.73) in the transition zone (corresponding to Figure 8b); (**c**) 2D Evaluation of a point (2.83, 2.77) inside the hazard zone (corresponding to Figure 8c).

In order to scrutinize the degrees of danger, the right-hand 3D plots in Figure 8 were converted to 2D plots, as shown in Figure 9. Figure 9a, which represents the case of ‘the point outside the hazard zone’, contains sporadic data points that are greater than a hazard index of 0, while having a large portion of points equal to a hazard index of 0. This trend in the hazard index increases as the subject moves towards the hazard area, as shown in the cases of ‘the point inside the hazard envelope but outside the hazard core (Figure 9b) and ‘the point inside the hazard core’ (Figure 9b). When the subject was estimated to be in the hazard core, the corresponding plot contained a large portion of points that were equal to or greater than a hazard index of 0.5.

In sum, the graphs indicate that higher scores were observed more frequently as the subject moved from outside of the hazard zone to inside the hazard envelope and from inside the hazard envelope to the hazard core. It is important to note that the analysis was based on a probabilistic estimation of the subject’s location, and it inherently contained probabilistic errors (as the standard deviation of the tracking system was used for error quantification). For the case described in Figure 9a, this probabilistic error produced 8% of the estimations to have discrete hazard indices higher than 0.5. This was reasonable because the position estimation (3.28, 0.37) was close to the transition boundary in which a small error could result in a non-zero hazard index. For the other cases described in Figure 9b,c, similar observations regarding the effect of the probabilistic assessment were found.

So far, this paper has introduced the assessment of each point based on the developed models and equations. Because each of these (*x*, *y*) points were assessed probabilistically, each had its own likelihood of occurrence at a rate of one per the number of data points, which in this case was 1/3000. After taking into account this likelihood for each datum point, the safety index was computed as shown in Equation (6). Note that the description made in this context focused on the three points selected; however, the safety evaluation system processed all of the estimated points—in this example, the estimated points were the points illustrated in Figure 7—and yielded the safety performance indices assessed for the points.

**Figure 10:**
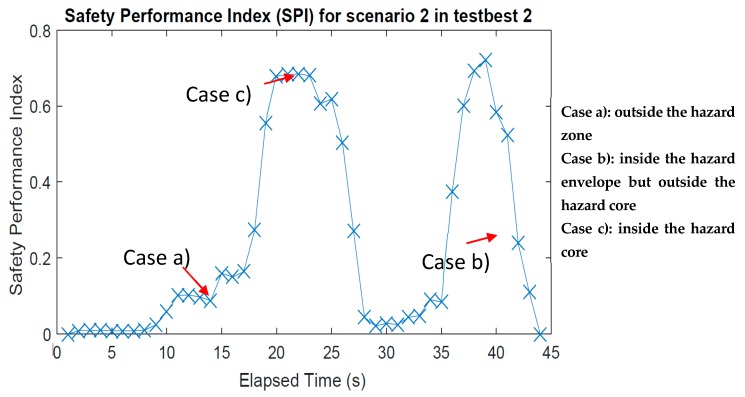
Safety performance index for Scenario 2 of Testbed 2.

Figure 10 presents the results of the ZBSR analysis for Scenario 2 of Testbed 2 on the safety performance index, which well-represented the safety conditions of the test subject in a probabilistic manner. Scrutiny of the data in Figure 10 revealed the following.

- The subject was in the safe area for about 10 s.
- From approximately 10 s, the subject was exposed to a low hazard level.
- From approximately 17 s, the hazard level increased sharply (spiked).
- The subject was detected as staying in the hazard zone from approximately 20 s to 25 s.
- From approximately 25 s, the hazard level dropped to almost zero at approximately 28 s.
- The subject was detected again in the hazardous zone, and the hazard level spikes up from approximately 35 s.
- The subject was detected to stay in the hazard from approximately 36 s to 40 s.
- From approximately 40 s, the hazard level dropped, and the safety hazard condition disappeared.

These summaries represent the actual movement reasonably well (i.e., ground truth) of the subject simulated in Scenario 2 in Testbed 2. When generating the safety performance index (SPI), these details were compiled into one single safety index as described in Equation (6). This quantification was important, and varied from the conventional method. The results not only described the behavioral phenomena of the subject but also quantified the safety performance of the subject, based on the given hazards and their associated modeling information.

**Figure 11:**
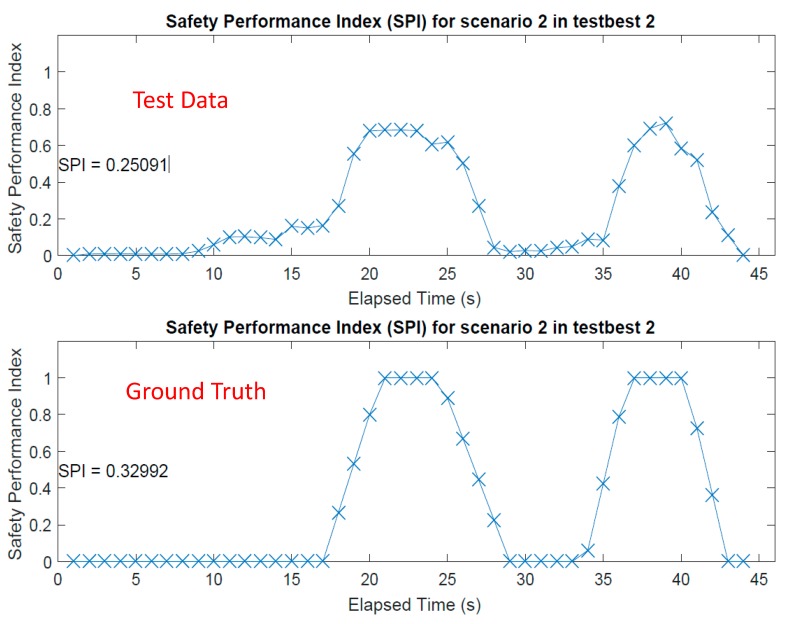
Comparison of safety performance indices for test data and ground truth.

To compare the safety performance index (SPI) resulting from the test data with that resulting from the ground truth, the same analysis was conducted on the ground truth data set. Figure 11 plots the SPI in a compact scale for each of the two cases, the test case and the ground truth case. Overall, the SPI of the test case seemed capable of reflecting the safety conditions of the subject as it represented relatively well the SPI trend. One of the intriguing findings from observation of the results was that the SPI of the test data underestimated the safety evaluation of the subject when compared to SPI based on ground truth data. Although this was not a desirable observation, it was inevitable because of the uncertainty of the associated parameters. The difference between the two SPIs could be partially explained by the sample data from Figure 8. Figure 8c shows scattered data points that are extracted from a given position data set. These scattered points indicate possible locations with unique weights assigned to them. In this case, because it was a probabilistic approach with the scattered points, the procedure not only used points within the core but also used points in the envelope area as well as in the safe area. However, in the case when using the ground truth, because it was a deterministic measure, the SPI was computed to be 1.0. As a result, the lower SPI by the system was inevitable: the SPI from the test data set was 0.251 critical safety incident per day for the test time period, and that from the ground truth was 0.330 critical safety incident per day. 

Another important observation, from analyzing certain segments in Figure 11, was that when the subject was near the boundary of a core zone (for times 20–25 s and 35–40 s), the automated safety evaluation approach, as compared to ground truths, underestimated the SPI. When the subject was near the boundary of an envelope zone (for times 10–15 s and 28–33 s), the automated approach overestimated the SPI. This observation was reasonable because the study used a tracking system that offered 1.5-m accuracy, which resulted in probabilistic errors. By using a more accurate tracking system or a dense system network, this issue of underestimation or overestimation could be reduced. 

Table 1 compares results from the test data and the corresponding ground truth data. Certain cases—including Scenarios 1 and 4 in Testbed 1 and Scenario 3 in Testbed 3—that simulate a safe situation but are located near the hazard envelope, led to a fairly accurate estimation of the safety condition as they produced errors of 0.0024 and 0.009 critical safety incident per day. This was because the differences between the SPIs of the ground truths and that of the test data sets were small. Other cases in which certain unsafe movements were observed resulted in similar findings, as discussed above. Note that the SPI values were based on the duration of the data collection, so the SPI values continuously changed with the data feed in the analysis (Figure 10 and Figure 11). For most cases, aggregated SPI values of the test data were lower than those for ground truth (Figure 11). Based on previous observations (i.e., underestimation near the core, which was discussed in detail with Figure 8c), this was expected because the test cases involved interactions with the hazard core, which was the case when underestimation was observed.

## 6. Conclusions

The construction industry has been suffering from inefficient methods of quantifying safety-related hazards with limited resources. To overcome this challenge, this study developed a sensor-based method by establishing a framework for an automated safety monitoring system, and presented a new analytical and computational method to evaluate the safety performance of workers by using a ZBSR model. To assess the performance of the developed model, two sets of experimental studies were conducted at construction sites.

The experimental studies assessed the ability of the ZBSR model for quantifying the safety performance index for workers with respect to pre-identified and registered hazards. Various hazards could be defined and updated differently based on their work duration; however, this was out of the scope of this study, and details can be found in a previous paper [12]. The various test scenarios and setups simulated diverse conditions, which varied the conditions of the parameters that affected the quantification of the safety index. For scenarios 1, 3, and 4 in testbed 1, and scenario 3 in testbed 3, errors were small, as none exceeded a 5% (0.045). Because of the nature of probability, the probabilistic evaluations showed slight underestimations for scenarios 2 and 5 in testbed 1, and scenarios 1 and 2 in testbed 2, with errors of 0.107, 0.113, 0.099, and 0.079 critical safety incidents per day, respectively. However, they represented the overall safety performance of a subject well, and will be improved if more accurate tracking is achieved. The test results showed clear evidence of the model’s capability in capturing the safety conditions of workers with respect to nearby hazards, based on location data from the tracking system. Such a capability to quantify the safety performance of workers provides unprecedented levels of information to the project/site manager. This information can be useful for daily safety trainings as well as for real-time warnings to reduce site risks.

The approach is advantageous over conventional methods because it can offer an impartial, automatic (or semi-automatic), and continuous job safety analysis, as well as a job-safety plan, thus eliminating problems related to workers’ safety stemming from a lack of understanding of the safety performance or the behavior of individual workers. Despite these advantages, this method is not yet to replace the current practice of safety inspections because the current safety site inspections and the proposed method of safety analysis address different aspects of safety concerns.

Although methodological and procedural developments were conducted in the research, the approach has a few limitations, which may be investigated in future research. First, the study relied on a tracking system that has a known accuracy level that was used as an input to the ZBSR analysis model. Second, the ZBSR model was limited to handling certain types of hazards that were defined by using geometric information for quantification purposes. Third, for more precise quantification of safety performance, the customization of workers in a parametric manner may be needed to account for skilled workers, as well as workers who are fully aware of a specific hazard and need to operate nearby the hazard. Fourth, because of the scope defined in the research, the evaluation of the safety performance index was not a reflection of safety evaluations for all types of safety issues on site, but was limited to those related to hazard zones. Last, but foremost, the purpose of ZBSR was to capture near-miss events and to quantify their risk levels in order to better understand the potential risks to workers when they are on site. However, most of the tested site data were based on simulated scenarios to validate the proposed theoretical approach. Thus, a case study with trade workers at a construction project would be necessary for a real-world validation. Furthermore, future study can explore each of the parameters in detail to refine their mathematical models and add additional parameters to add site and person dependent characteristics.

## Figures and Tables

**Table 1:** Comparison of the Aggregated Safety Performance Index for ground truth and test data.

Testbed	Scenario	SPI of Ground Truth	SPI of Test Data
1	1	0	0.0024
	2	0.328	0.221
	3	0.253	0.295
	4	0	0.028
	5	0.619	0.506
2	1	0.390	0.291
	2	0.330	0.251
	3	0	0.009
